# Integrative Multi-Omics Reveal Metabolic Reprogramming by Ketogenic Diet in Melanoma Xenografts

**DOI:** 10.3390/biom16071071

**Published:** 2026-07-22

**Authors:** Rohit Dnyansagar, Natalie Bordag, Rodolphe Poupardin, Julia Tevini, Victoria E. Stefan, Sophia Derdak, Martin Bilban, Nikolaus Fortelny, Barbara Kofler, Roland Lang, Daniela D. Weber

**Affiliations:** 1 Research Program for Receptor Biochemistry and Tumor Metabolism, Department of Pediatrics, University Hospital of the Paracelsus Medical Private University, 5020 Salzburg, Austria; r.dnyansagar@crcs.at (R.D.); j.tevini@salk.at (J.T.); v.stefan@salk.at (V.E.S.); b.kofler@salk.at (B.K.); 2 Department of Dermatology and Venereology, Medical University of Graz, 8010 Graz, Austria; n.bordag@medunigraz.at; 3 Cell Therapy Institute, Paracelsus Medical Private University, 5020 Salzburg, Austria; rodolphe.poupardin@pmu.ac.at; 4 Department of Biosciences and Medical Biology, University of Salzburg, 5020 Salzburg, Austria; 5 Core Facilities, Medical University of Vienna, 1090 Vienna, Austria; sophia.derdak@meduniwien.ac.at (S.D.); martin.bilban@meduniwien.ac.at (M.B.); 6 Department of Laboratory Medicine, Medical University of Vienna, 1090 Vienna, Austria; 7 Center for Tumor Biology and Immunology, Department of Biosciences and Medical Biology, University of Salzburg, 5020 Salzburg, Austria; nikolaus.fortelny@plus.ac.at; 8 Department of Dermatology and Allergology, University Hospital of the Paracelsus Medical Private University, 5020 Salzburg, Austria

**Keywords:** ketogenic diet, melanoma, multi-omics integration, cancer metabolism, metabolomics, transcriptomics

## Abstract

The ketogenic diet (KD) has demonstrated anti-proliferative effects across multiple tumor types, yet the underlying metabolic and transcriptomic mechanisms remain incompletely understood. This study employed integrated multi-omics analysis combining targeted metabolomics and RNA sequencing to elucidate KD-induced metabolic reprogramming in BRAF/NRAS wild-type, BRAF mutant, and NRAS mutant melanoma xenografts, which showed delayed tumor growth when treated with the KD. Despite pronounced metabolic and transcriptional heterogeneity across models with minimal overlap in individual KD-responsive genes, pathway-level analysis revealed convergent biological signatures. Using VIP score-based integration and supervised latent variable modeling (mixOmics DIABLO), we identified consistent KD-associated alterations in cancer-related pathways including the PI3K-Akt, MAPK, sphingolipid as well as HIF-1 signaling pathways. The KD enhanced sphingomyelin and ceramide levels and additionally induced transcriptional signatures, indicating increased ceramide synthesis and reduced ceramide breakdown. Moreover, the KD reduced transcript levels of genes encoding critical tumor regulators, including PI3K, AKT, HIF, MEK, and ERK. These findings demonstrate that despite metabolic and transcriptomic heterogeneity, the KD drives coordinated metabolic reprogramming at the pathway level, indicative of shifting lipid metabolism toward pro-apoptotic ceramides and attenuating key oncogenic signaling cascades. Our results provide insights into the KD’s anti-tumor efficacy and identify metabolic nodes amenable to therapeutic intervention in melanoma.

## 1. Introduction

Melanoma, a highly aggressive form of skin cancer, originates from melanocytes and is characterized by its significant genetic diversity and complex metabolic reprogramming [1]. Genomic studies have identified numerous alterations, including mutations, deletions, amplifications, translocations, and DNA methylation changes, contributing to its development and progression. Key driver mutations in genes such as BRAF, RAS, and NF1 classify melanomas into distinct genetic subtypes, which are crucial for guiding diagnosis and treatment in the era of precision medicine [2,3]. These driver alterations converge on aberrant activation of the mitogen-activated protein kinase/extracellular signal-regulated kinase (MAPK/ERK) signaling cascade, promoting uncontrolled proliferation and providing a rationale for targeted inhibition, including mitogen-activated protein kinase kinase (MEK) inhibitors and agents targeting the phosphoinositide 3-kinase/protein kinase B/mammalian target of rapamycin (PI3K/AKT/mTOR) pathway [3]. Despite substantial clinical advances achieved with targeted therapies, the development of acquired resistance remains a major obstacle in melanoma management. Immune checkpoint blockade, including anti-CTLA-4 and anti-PD-1 antibodies, has likewise transformed the therapeutic landscape. However, durable responses are not observed in all patients [4], underscoring the need for improved and combinatorial treatment strategies.

The classic Warburg effect, a shift from oxidative phosphorylation (OXPHOS) to glycolysis despite oxygen availability, is a recognized metabolic alteration of tumor cells [5]. However, the high metabolic flexibility of melanoma extends beyond this paradigm, allowing tumor cells to adapt and utilize various fuel sources to support their growth, progression, and metastasis [6]. Metabolic flux profiling revealed that melanoma cells maintain elevated glycolytic rates alongside functional tricarboxylic acid (TCA) cycle activity even under hypoxia [7]. Moreover, individual patient-derived melanomas and melanoma cell lines displayed variable metabolic signatures ranging from predominantly glycolytic to highly oxidative phenotypes [8,9,10]. Beyond glucose, glutamine serves as an anaplerotic substrate feeding the TCA cycle and supports fatty acid (FA) synthesis through reverse TCA flux, enabling metabolic adaptation to hypoxia [7,11]. Dysregulated lipid metabolism, particularly elevated fatty acid synthase activity driven by SREBP1c activation, supports the rapid proliferation of melanoma cells [12]. Melanoma cells undergo dynamic lipid remodeling of membrane composition during progression, which influences melanoma cell plasticity and aggressiveness [13]. Furthermore, increased fatty acid oxidation associates with drug resistance, metastatic progression, and poor prognosis [14,15,16]. In summary, melanomas present flexible, heterogeneous metabolic signatures encompassing glycolytic, glutaminolytic, and lipogenic/lipolytic phenotypes. The intricate metabolic interactions between cancer cells and their microenvironment further influence melanoma progression and immune response [17].

Given the metabolic vulnerabilities of melanoma, dietary interventions have emerged as a promising complementary approach to conventional cancer therapies [18,19]. The concept that diet can influence cancer treatment outcomes is gaining traction, with growing insights into how nutrient supply impacts tumor development and therapeutic responses. However, the effectiveness of dietary modulation is highly dependent on the specific characteristics of the cancer, including its tissue of origin, microenvironment, and genetics, necessitating a precision nutrition approach [18]. Dietary interventions can potentially limit nutrient availability for cancer cells, affecting their interaction with the stroma and reactivating anti-tumor immune responses, although they may also deprive immune cells of essential nutrients [20].

Among the dietary strategies, the ketogenic diet (KD), a high-fat, low-carbohydrate, adequate-protein diet, has shown potential in cancer therapy by exploiting the reprogrammed metabolism of cancer cells [21]. Preclinical studies have demonstrated anti-tumor effects of KDs, often when used as an adjuvant to chemotherapy and radiotherapy [21,22]. In addition, experimental models indicate that the KD modulates the tumor microenvironment (TME) and may augment immunotherapy responses by promoting the activation and infiltration of cytotoxic immune populations, including CD8^+^ T cells, M1-polarized macrophages, and natural killer cells, while reducing immunosuppressive subsets such as regulatory CD4^+^ T cells, M2-like macrophages, and myeloid-derived suppressor cells [23]. The KD triggers the production of ketone bodies, such as beta-hydroxybutyrate (BHB) and acetoacetate (AcAc), which can serve as an alternative energy source for normal cells and immune cells, while potentially creating an unfavorable metabolic environment for cancer cells [21,22]. The mechanisms underlying the anti-tumor effects of KDs are diverse and may involve not only the direct impact of ketone bodies and low blood glucose and insulin levels, but also broader systemic effects that can cooperate with specific therapies [18,21].

To gain a comprehensive understanding of melanoma’s biology and identify novel therapeutic targets, multi-omics integration of metabolomics and transcriptomics is becoming critical. This approach provides a systems-level perspective by combining the global assessment of cellular function from metabolomics with the gene expression profiles revealed from transcriptomics [24]. Integrating these data types can uncover key metabolic alterations and aberrantly expressed enzymes, enabling biomarker and therapeutic target identification [25].

Recently, we found that the KD effectively reduced tumor growth in athymic mice bearing genetically and metabolically heterogeneous human melanoma xenografts [26]. Our prior targeted analysis of plasma and tumor metabolomes revealed distinct changes in amino acid and lipid metabolism, notably reducing the levels of alpha-aminoadipic acid (alpha-AAA), a biomarker of cancer, to levels observed in tumor-free mice and also decreased the concentration of alpha-AAA in tumors. Moreover, the KD increased the sphingomyelin (SM) levels in plasma and the hydroxylation of SMs and acylcarnitines in tumors [26]. Building upon these metabolic observations, the present study newly generated mRNA sequencing (RNA-Seq) data from these identical xenograft tumors. This current work aims to extend our molecular understanding by integrating these transcriptomic profiles with the previously characterized metabolomic datasets [26], thereby providing novel insights into the coordinated molecular reprogramming underlying KD’s effects in melanoma.

## 2. Materials and Methods

### 2.1. Melanoma Xenografts

The metabolomics dataset and the xenograft tumor material utilized for the RNA sequencing analysis in this study originate from a previously published study [26] performed in accordance with the Salzburg Animal Care and Use Committee (study approval no. 20901-TVG/122/10-2018). Animals were maintained under specific pathogen-free conditions and care conformed to the Austria Act on Animal Experimentation. Mice had ad libitum access to water and chow.

Melanoma xenograft-bearing mice were established and treated as previously published [26]. In brief, the human melanoma cell lines A375, WM47, WM3311, and WM3000 were used to establish melanoma xenografts in 5- to 7-week-old female CD-1 nude mice (Charles River, Sulzfeld, Germany). Once the tumor volume reached 100 mm^3^, mice were equally assigned to the standard diet (SD) or a long-chain triglyceride-based ketogenic diet (KD) (Ssniff-Spezialdiäten, Soest, Germany) (n = 10–13 per group). All diets were given ad libitum. Detailed information on the diet composition and energy content is provided in Supplementary Table S1. WM47 and WM3311 melanoma-bearing mice were euthanized once the tumor size reached ~1000 mm^3^, whereas A375 and WM3000 melanoma-bearing mice were euthanized once the tumor size reached 10% of net body weight (2000–2500 mm^3^). Mice that approached >20% net body weight loss were euthanized immediately regardless of tumor size [26]. Once tumors reached humane endpoints, tumor tissue was harvested, snap frozen in liquid nitrogen, and stored at −80 °C for metabolomics and bulk RNA-Seq.

Physiological characterization from our prior work confirmed that KD-fed mice achieved a robust state of nutritional ketosis, evidenced by an average 525% increase in plasma beta-hydroxybutyrate (BHB) levels and an average 31% reduction in blood glucose (see Figure 2 in [26]). While the KD had heterogeneous effects on body weight across models, with some showing weight loss (WM47, WM3311) and others stable weight (A375, WM3000), our previous analysis found no significant correlation between tumor volume and body weight (Figure 1N,O in [26]), concluding that the observed anti-tumor effects of the KD occurred independently of body weight changes. This evidence, coupled with stable or increased body weight in some KD-treated groups, indicates that the observed tumor growth delay and subsequent multi-omics alterations are not primarily attributable to caloric restriction [26].

### 2.2. Targeted Metabolomics

Targeted metabolic profiling of xenograft samples was performed using the MxP^®^ Quant 500 Kit combined with the UHPLC-MS/MS-based acylcarnitine assay (Biocrates Life Sciences, Innsbruck, Austria) according to the manufacturer’s instructions and as previously described (n = 7–13 per group) [26,27].

Raw data were exported and quantified using the MetIDQ^TM^ software version 8.7.1 (Biocrates Life Sciences, Innsbruck, Austria). Quality control sample-based data normalization based on QC2 was performed to minimize the variation of analyses.

### 2.3. RNA Extraction and Sequencing

RNA was extracted from A375, WM47, WM3311, and WM3000 xenograft tissue using the AllPrep DNA/RNA/Protein Mini Kit (Qiagen, Germantown, MD, USA) according to the manufacturer’s instructions (n = 7–9 per group). Sequencing libraries were prepared at the Genomics Core Facility of the Medical University of Vienna using the NEBNext Poly(A) mRNA Magnetic Isolation Module, and the NEBNext UltraTM II Directional RNA Library Prep Kit for Illumina according to the manufacturer’s protocol (New England Biolabs, Ipswich, MA, USA). The libraries were QC-checked on a Bioanalyzer 2100 (Agilent, Santa Clara, CA, USA) using a high sensitivity DNA kit for correct insert size, and quantified using a Qubit dsDNA HS Assay (Invitrogen, Eugene, OR, USA). The pooled libraries were sequenced on two flow cells of a NextSeq500 instrument (Illumina, San Diego, CA, USA) in 1 × 75 bp single-end sequencing mode. Per sample, an average of 25 million reads were generated. Reads in a fastq format were generated using the Illumina bcl2fastq command line tool (v2.19.1.403) including trimming of the sequencing adapters. Since the samples were human xenografts inoculated in mice, an additional stringent pre-alignment was performed where all of the reads in fastq format were aligned to the murine reference genome version GRCm38/mm10 using STAR aligner version 2.6.1a in 2-pass mode [28], in order to computationally remove reads that originated from the mouse portion in the tissue sample. Reads that remained unmapped in the pre-alignment step were recovered and aligned to the human reference genome version GRCh38 with Gencode 29 annotations using STAR aligner version 2.6.1a in 2-pass mode. The raw reads per gene were counted with STAR [28]. The differential gene expression was calculated using DESeq2 version 1.50.2 [29], which normalizes data to account for sequencing depth and sample differences. The *p*-values were adjusted for multiple testing using the Benjamini–Hochberg method to control false discovery rate (FDR) at 0.05. In addition, |log2 fold change (FC)| > 1 was considered to select differently expressed genes. The transcripts per million (TPM) were generated by RNA-Seq with expectation maximization (RSEM) [30]. Principal component analysis (PCA) was used to investigate the transcriptomic difference between the four different melanoma xenograft models.

### 2.4. Multi-Omics Data Integration

Data processing and statistical analysis were conducted using R (v4.5.0), with plots generated via ggplot2 (v4.0.2). For the integrative multi-omics analysis, matched metabolomics and transcriptomics samples (n = 7–9 per group) were utilized (Supplementary Table S2).

#### 2.4.1. Data Preprocessing

Data cleaning of the metabolomics data was performed by excluding metabolites with >20% missing values or values below the limit of detection (LOD) in all experimental groups. Thus, all metabolites with >80% of the concentration values above the LOD in at least one of the experimental groups were included for statistical analysis. The resulting values were further log-transformed (log10) to enable downstream analysis and multi-omics integration.

Univariate statistical analysis was performed using the MetaboAnalyst R package [31]. Briefly, input data were loaded as a concentration table and remaining missing values after the described data cleaning step were replaced by 1/5 of the minimum positive value of each variable. Differential metabolites between the KD and SD were determined for each xenograft model using two-sample t-tests. Metabolites with an FDR-adjusted *p*-value (FDR) < 0.05 were considered statistically significant.

The TPM-normalized transcriptomics data were further log10-transformed and preprocessed to reduce noise from infrequently detected genes by applying a presence-based filtering strategy prior to integration. Specifically, genes with >30% of zero counts within both the SD group and the KD group for each xenograft were excluded. Therefore, genes with expression values in ≥70% of SD- and KD-samples were included for downstream analyses. To handle remaining zero counts and prevent mathematical errors during log10-transformation, a standard pseudocount of 1 was uniformly added to all TPM values prior to log10-transformation (log10(TPM + 1)).

#### 2.4.2. VIP Score-Based Joint Pathway Analysis

To identify the discriminating metabolites and genes driving the distinction between the KD and SD samples, we utilized a supervised multivariate pipeline for each individual xenograft model (A375, WM47, WM3000, and WM3311). Partial least squares-discriminant analysis (PLS-DA) was performed on the filtered metabolome and transcriptome matrices using the mixOmics R package v6.28.0 [32]. For each data layer within a cohort, a two-component supervised PLS-DA model (ncomp = 2) was fitted to maximize the covariance between the omics block (X) and the categorical dietary variable (Y). Model performance and generalization power were assessed using a 5-fold cross-validation (CV) framework. Model predictive power was quantified via balanced error rates (BER) using the maximum distance (max.dist) metric to report cross-validated classification accuracy (1−BER). To address potential overfitting inherent to PLS-DA, model quality was further validated. Since mixOmics does not calculate cumulative predictive capacity (Q2) or support permutation testing, these parameters were determined using the ropls R package (v3.23). Specifically, we performed 7-fold CV to calculate Q2 and ran random permutation testing with 100 iterations. An empirical *p*-value (pQ2 < 0.05) confirmed model robustness against overfitting (Supplementary Table S4).

To isolate the key molecular drivers segregating the KD and SD groups, variable importance in projection (VIP) scores were calculated across the latent space components. The resulting metabolites and genes with VIP ≥ 1, which represents a feature-prioritization criterion rather than a formal statistical significance threshold, were subsequently subjected to joint pathway analysis using the human Kyoto Encyclopedia of Genes and Genomes (KEGG) pathway library in MetaboAnalyst 6.0 [33,34]. The input data for the joint pathway analysis were corresponding Human Metabolome Database (HMDB) IDs and Gene Symbols for metabolites and genes, respectively (Supplementary Table S5). Pathway database parameter settings were selected to use all pathways (integrated). Algorithm selection parameter settings were chosen to use enrichment analysis based on hypergeometric test, topology measure based on degree centrality, and integration method based on combined queries. Pathways were considered significant if FDR < 0.05.

#### 2.4.3. mixOmics DIABLO Data Integration

The preprocessed and log10-transformed transcriptomics and metabolomics data from all four xenografts (A375, WM47, WM3000, WM3311) were combined and submitted to DIABLO (Data Integration Analysis for Biomarker discovery using Latent cOmponents) (mixOmics R package v6.28.0) [32,35], a supervised multi-block sparse partial least squares-discriminant analysis (s)PLS-DA framework, specifying two blocks (transcriptomics, metabolomics) with a full design (correlation enforced between blocks).

Performance of the DIABLO model was assessed using repeated 5-fold cross-validation. BER decreased with the inclusion of additional components. For the metabolomics block, BER stabilized below 0.1 after 4–6 components, indicating strong discriminatory power. Transcriptomics showed higher BER (~0.3–0.4), suggesting weaker individual classification ability but complementary information when integrated. The fourth discriminant component (component 4) showed the lowest BER and provided the clearest separation between KD- and SD-samples across xenografts (Supplementary Figure S1A). Metabolite and gene loadings of component 4 were extracted and ranked by absolute weight.

To evaluate whether the DIABLO integration model captures generalized biological phenotypes or is skewed by a single xenograft model, a rigorous Leave-One-Xenograft-Out (LOXO) validation framework was performed. Specifically, we iteratively excluded one of the four distinct melanoma xenograft models at a time (A375, WM47, WM3000, or WM3311), retrained the supervised DIABLO model on the remaining three xenografts, and extracted the feature loading weights for latent component 1 across both the metabolomics and transcriptomics data blocks. Pearson correlation coefficients (r) were then computed to directly evaluate feature weight alignment between the master model (trained on all four xenografts) and each of the four subset models (Supplementary Table S7).

Consequently, joint pathway analysis was performed using the KEGG pathway library in MetaboAnalyst. The input data were filtered to only include metabolites and genes representing the top 50% positive and negative loadings. The input data included HMDB IDs and Gene Symbols for metabolites and genes, respectively (Supplementary Table S9). All other parameters in MetaboAnalyst 6.0 were kept the same as for joint pathway analysis using metabolites and genes with VIP ≥ 1.

KEGG pathway maps were generated from the KEGG database (www.kegg.jp) [36] and modified using the Pathview R package (v1.50.0) [37].

#### 2.4.4. Gene Biotype

The transcriptome analysis included several genes that were unannotated, for example, AC099778.1 and AC099684.1. These genes were also present as input to VIP calculation and as significant contributors to the latent components in the factor analysis. However, since there is no reliable information available regarding their functional roles, these unannotated genes were excluded from the joint pathway analysis. However, an attempt was made to gather more information about these database entries via BioMart [38]. The BioMart entries of each of these accession numbers were queried and gene biotype information acquired. The summary of the gene biotypes of unannotated accession numbers is given in Supplementary Figure S2.

### 2.5. Declaration on the Use of AI in the Writing Process

Text.Cortex was used to assist with manuscript preparation in the following ways: (1) generating an initial draft of the abstract and the conclusion from the results and discussion sections, respectively (2) editing for grammar, spelling, and punctuation, and (3) improving language clarity and readability. All AI-generated content was reviewed and approved by the authors for accuracy and appropriateness.

## 3. Results

### 3.1. Multi-Omics Integration Workflow and Differential Metabolite/Gene Expression Analysis

**Figure 1:**
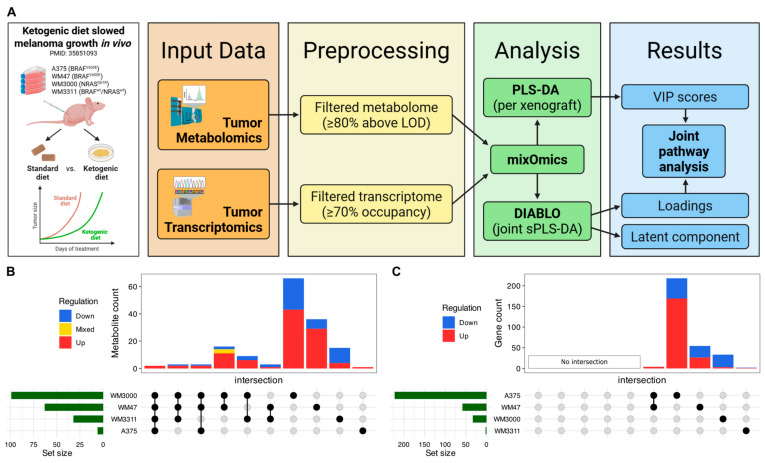
Overview of the multi-omics integration workflow and intersection analysis of ketogenic diet-associated alterations in the metabolome and transcriptome. (**A**) Schematic representation of the data integration workflow of metabolomics and transcriptomics. Created in BioRender. Weber, D. (2026) https://BioRender.com/6xhx9ov (accessed on 20 July 2026). (**B**,**C**) UpSet plots illustrate set intersections, i.e., which significant differential (**B**) metabolites and (**C**) genes were altered in several melanoma xenograft models or only within one model. For each xenograft, differential metabolites between the KD and SD were identified using a *t*-test with a significance threshold of FDR < 0.05 and differential genes were identified using DESeq2, applying FDR < 0.05 and |log2 fold change| > 1 as cutoffs (n = 7–9 per group). Blue indicates features downregulated by KD, red indicates features upregulated by KD, and yellow indicates features showing opposite regulation across xenograft models (i.e., upregulated in one model and downregulated in another).

Consistent with the previously reported anti-proliferative effects of KDs on a variety of tumor types, such as brain tumors, prostate cancer, pancreatic cancer, colon cancer, and neuroblastoma [21], our preclinical data showed that the KD significantly reduced the tumor growth of BRAF/NRAS wild-type (WM3311), BRAF^V600E^ (A375, WM47), and NRAS^Q61R^ (WM3000) melanoma xenografts in vivo (Figure 1A) [26]. Our previous targeted metabolomics analysis revealed significant systemic alterations in the plasma of melanoma-bearing mice under the KD, predominantly affecting amino acid and lipid metabolism [26]. In tumor tissue, the KD induced xenograft-specific metabolic changes, however, a subset of differential metabolites was shared across two, three, or all four melanoma models. For instance, the KD consistently upregulated beta-aminobutyric acid (BABA) and betaine consistently in all four xenografts (Figure 1B; Supplementary Table S3) [26].

Consistent with the previously reported metabolic heterogeneity of the four melanoma xenograft models [27] (Supplementary Figure S3A), the PCA of RNA-Seq-derived gene expression data revealed clear clustering according to the xenograft model, underscoring pronounced transcriptional differences between tumors (Supplementary Figure S3B). Notably, although A375 and WM47 xenografts both harbor the BRAF^V600E^ mutation, their global gene expression profiles separated distinctly, indicating substantial biological divergence beyond shared driver mutation. This transcriptomic and metabolic heterogeneity was independent of the final tumor volume (Supplementary Figure S3A,B).

Differential expression analysis comparing KD- and SD-treated tumors identified no common KD-responsive genes across all models, with only four overlapping genes between the two BRAF-mutant models A375 and WM47 (Figure 1C, Supplementary Table S3). Moreover, the extent of KD-induced transcriptional changes varied considerably between models: 218 genes were significantly altered in A375 tumors, 54 in WM47, and 33 in WM3000, whereas only two genes met the significance criteria in WM3311 tumors.

Given the pronounced heterogeneity in KD-associated transcriptional responses, we integrated metabolites and transcripts using two complementary multi-omics strategies. First, a PLS-DA-derived VIP score-based approach was applied within each xenograft, followed by joint pathway analysis to identify shared as well as distinct KD-induced pathway regulations between the xenografts. Second, a supervised latent variable framework (mixOmics DIABLO) was employed to jointly integrate the metabolomic and transcriptomic datasets across all xenografts (Figure 1A).

### 3.2. VIP Score-Based Joint Pathway Analysis Reveals Ketogenic Diet-Induced Shared Pathway-Level Alterations

**Figure 2:**
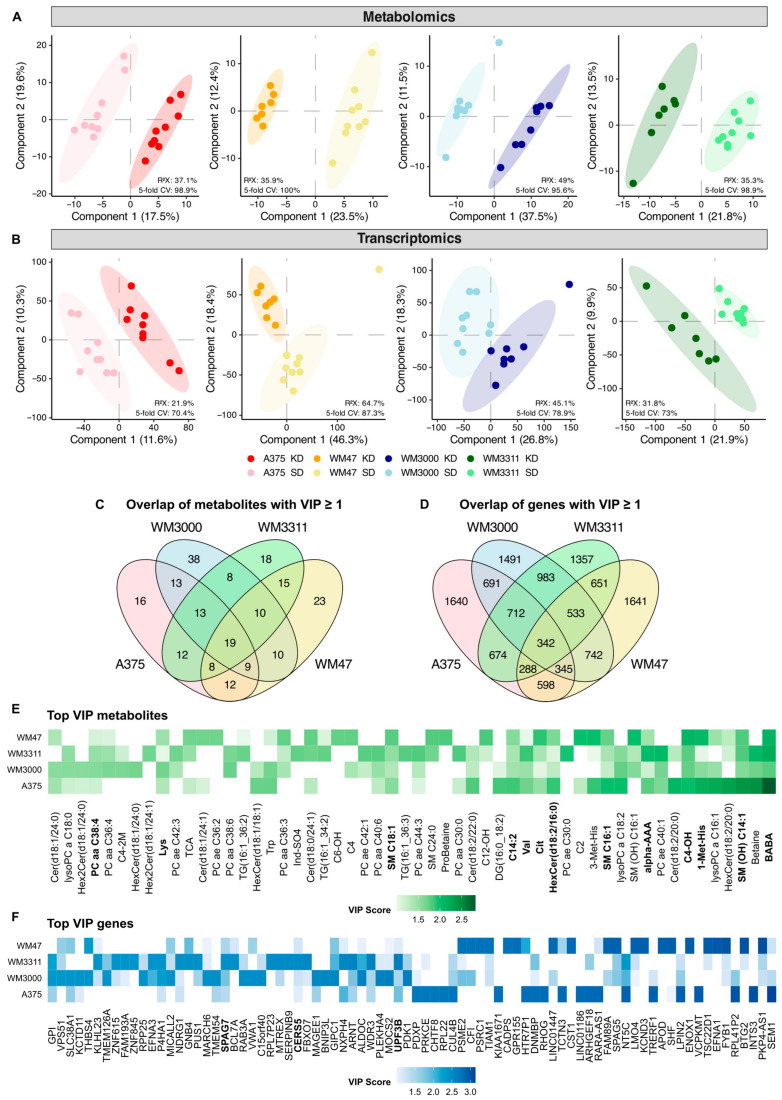
Ketogenic diet induces both specific and shared metabolic and transcriptomic changes in multiple melanoma xenografts. (**A**,**B**) Partial least squares-discriminant analysis (PLS-DA) plots illustrating 95% confidence ellipses of (**A**) metabolomics and (**B**) transcriptomics data obtained from A375, WM47, WM3000, and WM3311 melanoma xenograft tumors treated with the KD or SD (n = 7–9 per group). For each PLS-DA model, the cumulative R^2^X represents the total explained variation in the X-matrix. mixOmics 5-fold cross-validation (CV) was used to assess model accuracy. Calculation of the cumulative predictive capacity (Q2) and permutation testing (100 iterations, pQ2 < 0.05) was performed using ropls (Supplementary Table S4). (**C**,**D**) Venn diagram showing the overlap of (**C**) metabolites and (**D**) genes with a VIP score ≥ 1 across the four melanoma xenograft models. (**E**,**F**) Heatmap summarizing the top 20 (**E**) metabolites and (**F**) genes with a VIP score ≥ 1 for each of the four melanoma xenografts.

PLS-DA performed on individual xenografts revealed clear separation between the KD and SD tumor samples for both the metabolome and the transcriptome across all four melanoma models (Figure 2A,B). Fivefold CV in mixOmics revealed robust model predictive performance, yielding classification accuracies between 95.6% and 100% for the metabolomics data, and between 70.4% and 87.3% for the transcriptomics data (Figure 2A,B). Furthermore, CV and permutation testing using ropls verified the high predictive quality of these models, showing Q2 values between 0.61 and 0.93, with significant permutation *p*-values (pQ2 = 0.04 for WM3000 transcriptomics and pQ2 = 0.01 for all other models) (Supplementary Table S4).

The PLS-DA-derived VIP score, which ranks features based on their contribution to group separation, identified 110, 112, 134, and 109 metabolites, alongside 5291, 5142, 5845, and 5543 genes, with a VIP score ≥ 1 for the A375, WM47, WM3000, and WM3311 models, respectively (Supplementary Table S5). As shown in the Venn diagrams in Figure 2C,D, 19 metabolites and 342 genes with a VIP score ≥ 1 were shared across all four melanoma xenografts (Supplementary Table S5), representing a core molecular signature associated with KD-induced metabolic and transcriptomic reprogramming. Comparison of the top 20 VIP ≥ 1 features across models highlighted 13 key metabolites, including valine (Val), lysine (Lys), beta-aminobutyric acid (BABA), alphaα-AAA, 1-methylhistidine (1-Met-His), citrulline (Cit), the acylcarnitines C4-OH and C14:2, the trihexosylceramide Hex3Cer(d18:1/18:0), the phosphatidylcholine PC aa C38:4, and the sphingomyelins SM (OH) C14:1, SM C16:1, and SM C18:1. Additionally, three genes, namely *SPAG7*, *CERS5*, and *UPF3B*, maintained a VIP score ≥ 1 across all four models (Figure 2E,F). Despite these shared alterations, approximately 15% to 29% of the discriminating metabolites and 25% to 31% of the discriminating genes contributed uniquely to xenograft-specific separations (Figure 2C,D; Supplementary Table S5).

Overall, by identifying the molecular features driving KD-induced group separation based on their VIP scores, this analysis demonstrates that the KD triggers both distinct xenograft-specific responses and shared metabolic and transcriptomic adaptations across heterogeneous melanoma models.

To determine whether the identified discriminating features (VIP score ≥ 1) converge on identical biological pathways within each model, and to assess whether this results in shared or distinct pathway-level alterations across the different xenografts, we subjected the VIP ≥ 1 metabolites and genes to joint pathway analysis.

**Figure 3:**
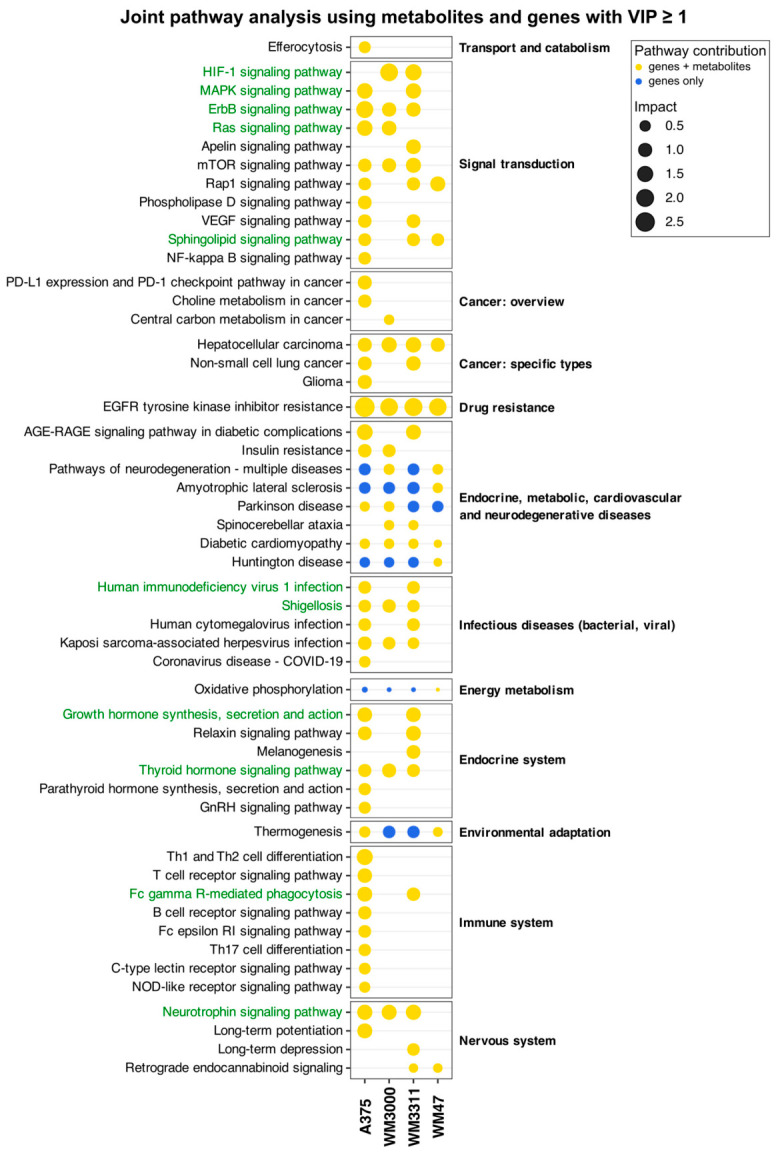
VIP score-based joint pathway analysis reveals ketogenic diet-induced shared pathway-level alterations across multiple melanoma xenografts. KEGG pathways identified through joint pathway analysis based on metabolites and genes with VIP ≥ 1. Symbol color indicates whether pathway enrichment was driven by combined gene and metabolite contributions (yellow) or by genes alone (blue). Symbol size reflects pathway impact. Significant pathways (FDR < 0.05) identified in at least one xenograft model with enrichment contributions from both genes and metabolites are shown (all signaling pathways are listed in Supplementary Table S6). Pathways are categorized according to the KEGG BRITE hierarchy. Pathway names highlighted in green indicate an overlap of pathways presented in Figure 3 and Figure 4C.

**Figure 4:**
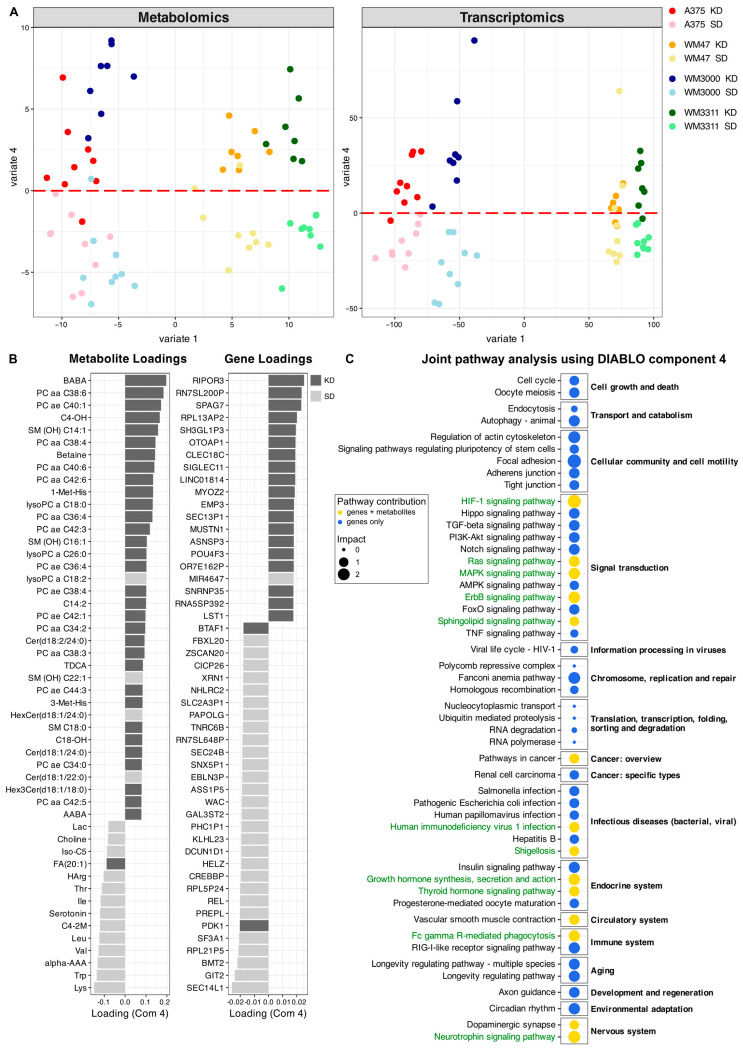
Functional characterization of the (s)PLS-DA-based multi-omics signature distinguishing ketogenic diet- from standard diet-treated tumors. (**A**) (s)PLS-DA plot showing component 1 (variate 1) and component 4 (variate 4) for the metabolomics and transcriptomics datasets (n = 7–9 per group). While components 1, 2, and 3 captured strong baseline biological differences among the four highly heterogeneous xenograft models, component 4 was identified as the primary dimension capturing the treatment-specific effect of the diet, indicated by the dashed red line. (**B**) Loading plots displaying the top 50 metabolites and genes contributing to DIABLO component 4. Positive and negative values represent coefficients of the loading vectors for each omics block. Features with larger absolute coefficients exert a stronger influence on the separation between KD- and SD-treated samples. Color coding indicates the group (KD or SD) contributing most strongly to the respective feature. (**C**) KEGG pathways identified through joint pathway analysis based on metabolites and genes representing the top 50% positive and negative DIABLO component 4 loadings, respectively. Symbol color indicates whether pathway enrichment was driven by combined gene and metabolite contributions (yellow) or by genes alone (blue). Symbol size reflects pathway impact. Significant pathways according to FDR < 0.05 are shown (all pathways in Supplementary Table S9) and classified by KEGG BRITE hierarchy. Pathway names highlighted in green indicate an overlap of pathways presented in Figure 3 and Figure 4C.

Joint pathway analysis of these discriminating metabolites and transcripts revealed significant pathway enrichment (FDR < 0.05) in 108, 43, 69, and 99 pathways for A375, WM47, WM3000, and WM3311 melanoma xenografts, respectively (Supplementary Table S6). Among these, 41, 11, 16, and 26 pathways were enriched through combined contributions from both genes and metabolites in A375, WM47, WM3000, and WM3311 xenografts, respectively, as depicted in Figure 3. Convergence at the pathway level across all four melanoma models was observed for several distinct categories: the cancer-specific pathway ‘Hepatocellular carcinoma’, the drug resistance pathway ‘EGFR tyrosine kinase inhibitor resistance’, the cardiovascular disease-related pathway ‘Diabetic cardiomyopathy’, and the neurodegenerative disease pathways ‘Pathways of neurodegeneration—multiple diseases’, ‘Amyotrophic lateral sclerosis’, ‘Parkinson disease’, and ‘Huntington disease’. Additionally, pathways related to energy metabolism and environmental adaptation, specifically ‘Oxidative phosphorylation’ and ‘Thermogenesis’, were consistently enriched across all models (Figure 4). Furthermore, pathways significantly enriched through joint metabolite and gene contributions in at least three of the four melanoma models included key signal transduction pathways, namely ‘ErbB signaling’, ‘mTOR signaling’, ‘Rap1 signaling’, and ‘Sphingolipid signaling’. This category also encompassed ‘Shigellosis’, ‘Kaposi sarcoma-associated herpesvirus infection’, ‘Thyroid hormone signaling’, and ‘Neurotrophin signaling’ (Figure 3). It should be noted that disease-related KEGG pathways, such as ‘shigellosis’, often reflect altered activity within shared molecular signaling modules rather than literal infectious disease biology.

Collectively, these findings from the VIP-based joint multi-omics pathway integration suggest that the KD modulates convergent biological pathways across diverse melanoma models, despite pronounced tumor heterogeneity.

### 3.3. Functional Characterization of the Ketogenic Diet-Associated (s)PLS-DA-Based Multi-Omics Signature

Complementary to the VIP score-based approach, we utilized DIABLO within the mixOmics framework. This supervised N-integration method extracts shared information across multiple data types measured on the same biological samples by selecting discriminative molecular features [35]. We assessed the classification performance of transcriptomic and metabolomic blocks across latent components by computing the balanced error rate using cross-validation (Supplementary Figure S1A). Integration of the metabolomic and transcriptomic datasets identified latent component 4, which distinctly separated KD- from SD-treated tumors, irrespective of xenograft origin (Figure 4A). The (s)PLS-DA score plots (Figure 4A) revealed slightly stronger group separation based on metabolomics compared to transcriptomics. A Pearson correlation of 0.78 between the variates of the two omics blocks for component 4 indicates strong concordance (Supplementary Figure S1B), suggesting that the biological signatures driving the separation between KD- and SD-treated tumors are consistent across both the metabolome and transcriptome. Furthermore, the LOXO validation framework confirmed that the DIABLO integration results are not driven by a single xenograft model, as the multi-omics signature remained highly preserved and structurally robust across all sub-iterations (r > 0.6) (Supplementary Table S7).

The loading plots in Figure 4B display the top 50 metabolite and gene loadings contributing to component 4 (Supplementary Table S8). Among the top 50 metabolite loadings, seven of the twelve differential metabolites shared across all four melanoma xenograft models were represented: BABA, betaine, Hex3Cer(d18:1/18:0), SM(OH) C14:1, C4-OH, alpha-AAA, and HArg. Furthermore, the KD-associated metabolite signature prominently included multiple sphingolipids (Cer, SM) and glycerophospholipids (PC, lyso-PC), highlighting a substantial contribution of lipid-related species to component 4.

The top 50 gene loadings included *CREBBP*, which encodes the cAMP-response element binding (CREB) binding protein (CREBBP or CBP). This transcriptional coactivator and histone acetyltransferase interacts with CREB to promote melanoma progression, metastasis, and cancer cell survival [39]. Although not statistically significant, *CREBBP* expression was lower in KD-treated WM3000 tumors compared to SD-treated tumors (FDR = 0.086, log2FC = −0.309), consistent with the direction of its gene loading. Another gene downregulated by the KD in WM3000 tumors and contributing to the KD-associated gene signature was *PDK1* (FDR = 0.002, log2FC = −0.695), encoding pyruvate dehydrogenase kinase 1, a critical metabolic regulator in melanoma. PDK1 is often overexpressed to drive aerobic glycolysis and support tumor growth [40,41].

To gain a functional understanding of the multi-omics integration, metabolites and genes representing the top 50% of positive and negative loadings were subjected to joint pathway analysis using KEGG. We identified 50 significantly enriched pathways (FDR < 0.05), of which 36 were enriched solely through gene contributions, while 14 were enriched through contributions from both genes and metabolites (Figure 4C, Supplementary Table S9). KD-associated transcriptomic and metabolomic changes induced alterations in several cancer-related pathways, including ‘PI3K-Akt signaling’ and ‘MAPK signaling’, both notably activated in melanoma (Figure 4C).

Moreover, within the pathways highlighted in Figure 4C, ‘shigellosis’, ‘sphingolipid signaling’, and ‘hypoxia-inducible factor 1 (HIF-1) signaling’ emerged as the top three pathways significantly enriched by KD-induced alterations when sorted by the number of contributing metabolites (Supplementary Table S9). Specifically, ‘shigellosis’ was enriched through contributions from 114 genes and 4 metabolites—leucine, isoleucine, diacylglycerol, and trihexosylceramide. ‘Sphingolipid signaling’ was enriched through 63 genes and the 3 metabolites SM, ceramide (CER), and diacylglycerol. Moreover, ‘HIF-1 signaling’ was enriched through 69 genes, including *PDK1* and *CREBBP*, and the 2 metabolites lactate and diacylglycerol. The respective metabolite and gene changes induced by the KD are represented in the corresponding KEGG maps of these pathways (Supplementary Figures S4–S6). Notably, all three pathways, ‘shigellosis’, ‘sphingolipid signaling’, and ‘HIF-1 signaling’, were interconnected with ‘MAPK signaling’, and both ‘sphingolipid signaling’ and ‘HIF-1 signaling’ were additionally interconnected with ‘PI3K-Akt signaling’ (Supplementary Figures S4–S8). This interconnectivity suggests strong crosstalk between these pathways and their collective connection to metabolic pathways hyperactivated in melanoma. Furthermore, ‘shigellosis’, ‘sphingolipid signaling’, and ‘HIF-1 signaling’ were also identified in the joint pathway analysis using metabolites and genes with VIP score ≥ 1 as input (Figure 3), reinforcing their prominence in KD-induced metabolic reprogramming.

The color-coded KEGG maps reveal that the KD induced transcriptional signatures indicating reduced activity of key regulators of melanoma tumorigenesis, including PI3K, AKT, HIF-1, MEK, and ERK (Supplementary Figures S4–S8), suggesting a potential link to melanoma proliferation. Moreover, within the sphingolipid signaling pathway, serine, SM, and CER species were increased by KD, suggesting enhanced sphingolipid synthesis. This finding is further supported by KD-induced transcript-level signatures suggestive of increased CER synthase (*CerS*) and decreased ceramidase (*CDase*). This metabolic shift may contribute to limiting the synthesis of sphingosine 1-phosphate (S1P), a tumor-promoting metabolite [42], and appears associated with favoring CER synthesis, which may contribute to the anti-tumor efficacy of KD.

## 4. Discussion

Previous work by our group and others has demonstrated that the KD substantially reshapes the metabolome in various disease contexts and samples, including blood, tumor cells, and CAR T cells, with metabolic changes extending far beyond elevated BHB levels and encompassing reduced insulin signaling and marked alterations in amino acid and lipid metabolism [26,43,44,45,46]. Beyond metabolic changes, emerging evidence indicates that the KD and BHB modulate gene expression. For instance, studies in glioma demonstrated that the KD could reverse tumor gene expression patterns toward those characteristic of non-malignant cells [47,48], with particular effects on genes encoding growth factor signal transduction components. Furthermore, BHB promotes transcriptional and epigenetic changes in CAR T cells, enhancing metabolic resilience through increased oxidative phosphorylation and augmented effector functions [46].

This study aimed to elucidate the metabolomic and transcriptomic basis of KD’s previously demonstrated anti-tumor effects in melanoma [26] through an integrated analysis of metabolomic and transcriptomic profiles in KD-treated tumors. However, multi-omics integration presents several well-known technical and analytical challenges [49], many of which were encountered in this study.

A fundamental limitation in multi-omics analyses arises from disparities in feature dimensionality across data layers. Metabolomic datasets typically encompass hundreds of distinct features (~500 metabolites), while transcriptomic datasets often contain tens of thousands of genes (~25,000–30,000). This substantial imbalance can bias integrative analyses toward the higher-dimensional omics layer. Consequently, a major concern arises in joint pathway analysis, where metabolites and genes serve as input features despite their unequal representation. We mitigated this limitation by not only reporting all significantly enriched pathways, but also quantifying the relative contributions of metabolites and genes to pathway enrichment. This stratified reporting enables a clear distinction between pathways driven primarily by transcriptomic changes and those where both omics layers substantively contribute. For identifying latent factors discriminating KD- and SD-treated groups, we employed the DIABLO framework from the mixOmics package [32,35]. This approach permits a layer-specific assessment of contributions, ensuring that each omics dataset informs the latent variables independently, thereby minimizing dimensionality-related bias.

Tool selection constitutes another significant analytical challenge in multi-omics research. A diverse landscape of computational platforms exists, with individual tools often optimized for specific data types or experimental designs [50]. Consequently, identifying the most suitable analytical tool for a given dataset typically requires iterative evaluation, consuming substantial time and computational resources. Functional annotation and pathway analysis further complicate integration efforts, as most biological databases are curated independently with limited cross-database consistency. Few analytical platforms seamlessly integrate information from multiple curated databases while maintaining reliability. In this study, we selected MetaboAnalyst with KEGG database integration, which offers robust, widely-validated pathway annotation [33]. However, we acknowledge inherent KEGG limitations, particularly regarding its ability to discriminate between closely related lipid species that differ only in their fatty acid composition.

Finally, a critical challenge in multi-omics research involves demonstrating the added analytical value of data integration, specifically identifying biological insights that emerge from combined analysis but would remain obscured in single-omics studies. In our prior metabolomic analysis, the KD induced alterations in glycine, serine, and threonine metabolism, lysine degradation, and arginine biosynthesis [26]. Notably, integrated metabolomic-transcriptomic analysis in this study revealed additional pathway alterations and interactions that substantially extend our understanding of KD’s effects on melanoma biology.

Multi-omics integration revealed that the KD substantially altered MAPK and PI3K-Akt signaling in melanoma xenografts. These pathways are constitutively activated in melanoma and are known to hyperactivate glycolytic metabolism through the upregulation of HIF-1 alpha and MYC [51,52]. Consistent with this, the KD reduced the transcript levels of critical pathway components, including PI3K, AKT, MEK, ERK, HIF-1, and MYC, indicating the attenuation of pro-proliferative signaling. Furthermore, the KD substantially altered HIF-1 signaling, which is deeply interconnected with both the MAPK and PI3K-Akt pathways, as indicated by decreased transcript levels of key glycolytic regulators including glucose transporter 1 (*GLUT1*) and hexokinase (*HK*). Although our targeted metabolomic analysis did not quantify glucose in tumor tissue, plasma glucose levels were significantly reduced in KD-treated melanoma-bearing mice [26], underscoring the functional impact of these transcriptomic changes.

Beyond these canonical oncogenic pathways, multi-omics integration revealed that the KD modulated specific metabolites and genes enriched within the KEGG ‘shigellosis’ pathway. However, consistent with a common challenge in KEGG annotation, disease-labeled pathways often reflect shared, generic inflammatory, or stress-signaling modules rather than direct pathogenic mechanisms. In this context, the ‘shigellosis’ enrichment is best interpreted as an annotation artifact, highlighting common signaling nodes rather than infectious disease biology. Specifically, our analysis indicates that MAPK signaling, a critical shared node, is highly interconnected within this pathway and was indeed modulated by KD. The observed changes in the ‘shigellosis’ pathway were primarily driven by KD-induced alterations in leucine, isoleucine, trihexosylceramide, and diacylglycerol. Notably, diacylglycerol is also a key component in HIF-1 and sphingolipid metabolism, further underscoring its role as a shared metabolic node influencing multiple pathways.

Multi-omics integration revealed substantial and biologically coherent KD-induced alterations in sphingolipid signaling. Sphingolipid dysregulation is a hallmark of melanoma progression; specifically, melanoma cells characteristically maintain low intracellular CER levels while accumulating tumor-promoting CER derivatives such as S1P and gangliosides [13,14,42]. This metabolic state is maintained through the selective downregulation of CER-synthesizing enzymes (e.g., CerS) and the upregulation of CER-degrading enzymes (e.g., CDase) during malignant transformation [42]. CERs function as critical signaling molecules responding to cellular stresses including DNA damage, nutrient deprivation, and oxidative stress [53,54], although their roles in cancer biology are context-dependent and sometimes divergent [55].

Our multi-omics analysis demonstrated that the KD increased CER levels and induced transcript-level signatures implicating upregulated expression of two key CER-producing enzymes: CerS, which catalyzes CER synthesis from sphingosine and fatty acids, and acid sphingomyelinase (A-SMase), which hydrolyzes SM to CER in the lysosomal compartment. These findings align with published observations that CerS6 expression is suppressed in melanoma cell lines and inversely correlates with metastatic potential [56], and that A-SMase expression inversely correlates with tumor stage in patient biopsies [57]. In contrast, the KD induced transcriptional signatures indicating downregulated CDase expression, encoding an enzyme responsible for CER breakdown. Notably, acid CDase is characteristically elevated in human melanoma cell lines and patient biopsies [58].

Although sphingosine and S1P were not measured directly in our targeted metabolomic approach, the integrated multi-omics analysis leads us to hypothesize that the KD may shift the balance toward CER accumulation over breakdown, thereby potentially limiting S1P production. This interpretation is supported by the observation that the KD induced transcriptional signatures indicating reduced MAPK and PI3K-Akt signaling, since S1P can activate these pathways through G-protein-coupled receptor signaling [59]. Furthermore, CERs have been shown to suppress pro-survival PI3K-Akt signaling by inhibiting AKT phosphorylation [60].

Beyond their signaling functions, CERs participate in multiple metabolic processes affecting lipid metabolism, oxidative phosphorylation, and glycolytic flux [61]. Elevated CER synthesis, which requires sphingosine and fatty acid substrates, suppresses de novo lipogenesis and fatty acid synthesis, thereby restricting lipid availability for membrane biogenesis and energy storage, both processes critical for cancer cell proliferation and survival [62]. High CER levels also compromise mitochondrial membrane integrity and function, thereby altering oxidative phosphorylation and ATP production. CERs additionally attenuate glycolytic metabolism by modulating key glycolytic enzymes and contributing to suppression of the Warburg effect [63]. Thus, the KD-induced upregulation of CERs likely exerts anti-tumor effects through both signaling and metabolic mechanisms, potentially creating an unfavorable metabolic microenvironment for tumor cell survival.

SMs represent another important sphingolipid class with critical roles in membrane lipid homeostasis during melanogenesis [13]. Comparative lipidomic analyses between melanocytes and melanoma cells, as well as between primary and metastatic melanomas, have revealed substantial alterations in membrane lipid composition associated with malignant transformation, including marked reductions in SMs [64]. This shift is mechanistically linked to the downregulation of SM synthase (SMS), the enzyme catalyzing SM production from CERs and phosphatidylcholines, with a consequent accumulation of glucosylceramides [65]. Low SMS expression is associated with adverse prognosis in metastatic melanoma [65]. Our analysis identified SMs among the ten metabolites consistently upregulated by the KD across all four xenografts. The KD-induced elevation of SMs in tumor tissue is reflected by positive KEGG loadings in the sphingolipid signaling pathway map. These findings corroborate our previous observations that despite heterogeneous lipid profiles across melanoma subtypes, the KD uniformly increases the plasma SM concentrations in melanoma-bearing mice [26]. Additionally, the KD increased the plasma ratio of SMs to both CERs and phosphatidylcholines, suggestive of enhanced SMS activity [62]. Notably, enriched sphingolipid metabolism was also detected in plasma from patients with pancreatobiliary cancer consuming a KD for two weeks [44].

A significant consideration when interpreting our findings is the experimental model employed. The melanoma xenografts were established in athymic CD-1 nude mice, which are immunodeficient due to the absence of functional T cells. Therefore, while the KD is known to influence immune responses in immunocompetent settings [23], our data cannot provide insights into T-cell mediated anti-tumor immunity, immune cell infiltration, or immune-modulating effects relevant to immunotherapy. Our findings, instead, focus on the direct metabolic and transcriptional alterations within the tumor cells detectable in this immunodeficient environment. Further research in immunocompetent models would be essential to elucidate the immune-mediated contributions of the KD to anti-cancer efficacy.

Another important limitation of this study is the tumor tissue collection strategy, which prioritized assessing the KD’s ability to delay tumor growth in our xenograft models [26]. This resulted in KD- and SD-treated tumors being harvested at different time points and tumor volumes. Consequently, our multi-omics analysis was performed on ‘survivor’ cells, meaning that observed metabolic and transcriptomic signatures might represent adaptive responses, rather than solely the direct anti-tumor effects of the KD. Due to the retrospective nature of this analysis, robust covariate control for tumor characteristics was not feasible. Future prospective studies should rigorously control these variables (e.g., by matching tumor volumes or fixed time points) to better delineate direct KD effects on tumor cells.

Collectively, our findings suggest that the KD may exert anti-tumor effects, at least in part, by modulating the characteristic dysregulation of sphingolipid signaling and metabolism in melanoma. Emerging preclinical and clinical evidence supports CERs as promising therapeutic agents for combination cancer therapy, compatible with chemotherapy, immunotherapy, targeted therapy, hormone therapy, anti-angiogenic therapy, sphingosine kinase inhibitors, and apoptosis modulators [61]. Given the demonstrated synergistic potential of a KD with multiple cancer treatment modalities including chemotherapy, radiotherapy, and immunotherapy, combining a KD with CER-based therapeutic strategies, potentially through CER-enriched KD formulations, represents an intriguing avenue for enhancing cancer therapy efficacy. However, such approaches require rigorous preclinical and clinical evaluation.

## 5. Conclusions

This integrated metabolomic-transcriptomic analysis elucidates molecular aspects of the KD’s anti-tumor effects in melanoma. Despite tumor heterogeneity, multi-omics integration identified changes in gene expression and metabolic profiles indicative of the reduced activity of MAPK, PI3K-Akt, and HIF-1 signaling networks and a shift in sphingolipid metabolism toward CER accumulation. These findings provide an evidential foundation for investigating combination therapies, such as a KD combined with CER-based approaches, to enhance anti-cancer efficacy. However, clinical translation requires rigorous validation through additional preclinical and clinical studies.

## Data Availability

All data that support the findings of this study are included in the article and its Supplementary Materials. Metabolomics data of samples from melanoma-bearing mice are available at the NIH Common Fund’s National Metabolomics Data Repository (NMDR) website, the Metabolomics Workbench, https://www.metabolomicsworkbench.org where they have been assigned Project ID PR001198. The data can be accessed directly via its Project DOI: 10.21228/M8VD7F. This work was supported by NIH grant U2C-DK119886. Raw RNA-Seq data files for this study have been deposited in the European Nucleotide Archive (ENA) at EMBL-EBI under the accession number PRJEB110273 (https://www.ebi.ac.uk/ena/browser/view/PRJEB110273 (accessed on 20 July 2026)). The RNA-Seq count table has been deposited at ArrayExpress under the accession number E-MTAB-16979 (https://www.ebi.ac.uk/biostudies/ArrayExpress/studies/E-MTAB-16979 (accessed on 20 July 2026)). The R code used in this study is available on GitHub (https://github.com/koflergroupketo/KetoAnalysisTools (accessed on 20 July 2026)).

## Supplementary Materials

The following supporting information can be downloaded at: https://www.mdpi.com/article/10.3390/biom16071071/s1. Figure S1: Assessment of DIABLO integration performance and concordance; Figure S2: Gene biotype composition of unannotated transcriptomic features; Figure S3: Melanoma xenografts differ distinctly in their metabolome and transcriptome; Figure S4: Integration of metabolomic and transcriptomic DIABLO loadings within the human Shigellosis pathway; Figure S5: Integration of metabolomic and transcriptomic DIABLO loadings within the human Sphingolipid signaling pathway; Figure S6: Integration of metabolomic and transcriptomic DIABLO loadings within the human HIF-1 signaling pathway; Figure S7: Integration of metabolomic and transcriptomic DIABLO loadings within the human MAPK signaling pathway; Figure S8: Integration of metabolomic and transcriptomic DIABLO loadings within the human PI3K-Akt signaling pathway. Table S1: Composition of standard and ketogenic diet; Table S2: Overview of samples used for integrative metabolomics and transcriptomics analysis; Table S3: Overview of differential metabolites and differentially expressed genes in tumor tissue of melanoma-bearing mice treated with standard or ketogenic diet. Related to Figure 1B,C; Table S4: Cross-validation and permutation testing results using the ropls R package; Table S5: Metabolites and genes with VIP score ≥ 1 derived from PLS-DA. Related to Figure 2 and Figure 3; Table S6: Joint pathway analysis results for each xenograft model using metabolites and genes with VIP score ≥ 1. Related to Figure 3; Table S7: Stability matrix of the Leave-One-Xenograft-Out (LOXO) validation framework; Table S8: Metabolite and gene loadings of DIABLO component 1–10. Related to Figure 4B; Table S9: Joint pathway analysis results using metabolites and genes representing the top 50% positive and negative loadings from DIABLO analysis. Related to Figure 4C.

## Abbreviations

The following abbreviations are used in this manuscript:

alpha-AAA	Alpha-Aminoadipic Acid
A-SMase	Acid Sphingomyelinase
AKT	Protein Kinase B
BABA	Beta-Aminobutyric Acid
BHB	Beta-Hydroxybutyrate
CDase	Ceramidase
CER	Ceramide
CerS	Ceramide Synthase
Cit	Citrulline
DIABLO	Data Integration Analysis for Biomarker Discovery Using Latent Components
ERK	Extracellular Signal-Regulated Kinase
FC	Fold Change
HArg	Homoarginine
HDAC	Histone Deacetylase
HIF-1	Hypoxia-Inducible Factor 1
KD	Ketogenic Diet
KEGG	Kyoto Encyclopedia of Genes and Genomes
FDR	False Discovery Rate
mTOR	Mammalian Target of Rapamycin
MEK	Mitogen-Activated Extracellular Signal-Regulated Kinase
OXPHOS	Oxidative Phosphorylation
FDR	FDR-Adjusted *p*-Value
PCA	Principal Component Analysis
PI3K	Phosphoinositide 3-Kinase
PLS-DA	Partial Least Squares-Discriminant Analysis
RNA-Seq	mRNA Sequencing
S1P	Sphingosine 1-Phosphate
SD	Standard Diet
SM	Sphingomyelin
(s)PLS-DA	Sparse Partial Least Squares-Discriminant Analysis
TCA cycle	Tricarboxylic Acid Cycle
TME	Tumor Microenvironment
TPM	TPM
VIP	Variable Importance in Projection
