# Supplementary material for: Integrative Multi-Omics Reveal Metabolic Reprogramming by Ketogenic Diet in Melanoma Xenografts
Source: Biomolecules. 2026 Jul 22;16(7):1071. doi: 10.3390/biom16071071 (PMC13407059; doi:10.3390/biom16071071)
Supplement: Supplementary file 1 [file biomolecules-16-01071-s001.zip › 20260710_Supplementary Figures_REVISED.pdf]

Supplementary Data

Integrative Multi-Omics Reveal Metabolic Reprogramming by Ketogenic Diet in Melanoma Xenografts

Rohit Dnyansagar [1], Natalie Bordag [2], Rodolphe Poupardin [3], Julia Tevini [1], Victoria E. Stefan [1,4], Sophia Derdak [5], Martin Bilban [5,6], Nikolaus Fortelny [7], Barbara Kofler [1], Roland Lang [8,*,†] and Daniela D. Weber [1,*,†]

[1] Research Program for Receptor Biochemistry and Tumor Metabolism, Department of Pediatrics, University Hospital of the Paracelsus Medical Private University, 5020 Salzburg, Austria; r.dnyansagar@crcs.at (R.D.); j.tevini@salk.at (J.T.); v.stefan@salk.at (V.E.S.); b.kofler@salk.at (B.K.)

[2] Department of Dermatology and Venereology, Medical University of Graz, 8010 Graz, Austria;
n.bordag@medunigraz.at

[3] Cell Therapy Institute, Paracelsus Medical Private University, 5020 Salzburg, Austria; rodolphe.poupardin@pmu.ac.at

[4] Department of Biosciences and Medical Biology, University of Salzburg, 5020 Salzburg, Austria

[5] Core Facilities, Medical University of Vienna, 1090 Vienna, Austria; sophia.derdak@meduniwien.ac.at (S.D.); martin.bilban@meduniwien.ac.at (M.B.)

[6] Department of Laboratory Medicine, Medical University of Vienna, 1090 Vienna, Austria

[7] Center for Tumor Biology and Immunology, Department of Biosciences and Medical Biology,
University of Salzburg, 5020 Salzburg, Austria; nikolaus.fortelny@plus.ac.at

[8] Department of Dermatology and Allergology, University Hospital of the Paracelsus Medical Private
University, 5020 Salzburg, Austria

* Correspondence: r.lang@salk.at (R.L.); d.weber@salk.at (D.D.W.)

† These authors contributed equally to this work.

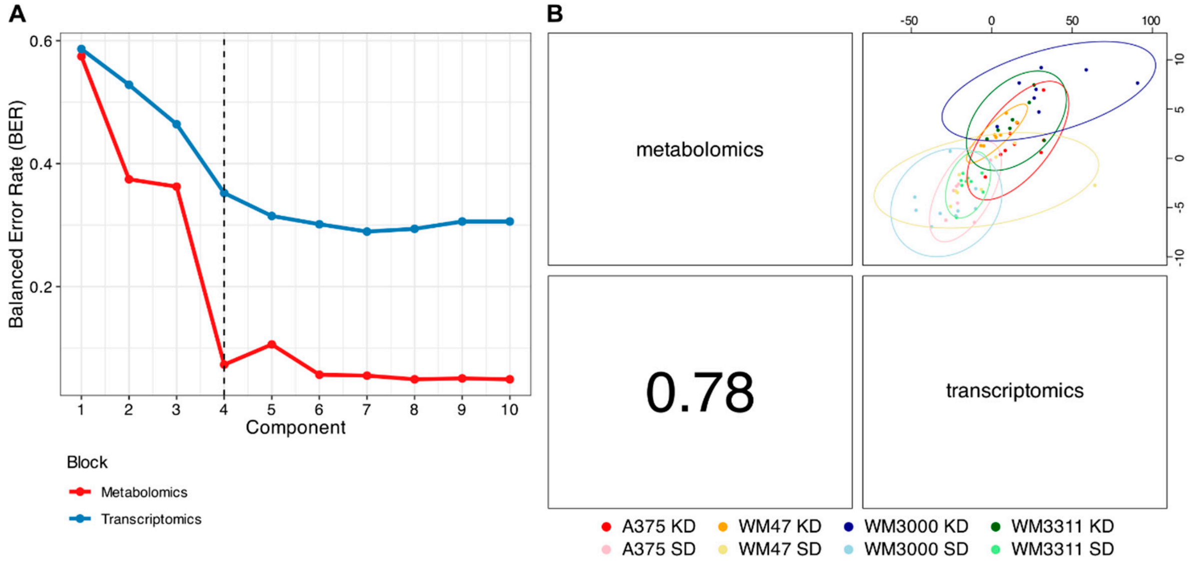

Supplementary Figure S1. Assessment of DIABLO integration performance and concordance. (**A**) Balanced error rate (BER) across latent components in DIABLO analysis. The curve represents a distance metric used in the DIABLO framework (centroid distance). The dashed line indicates the selected component 4, which optimally discriminates between KD- and SD-treated tumors. (**B**) Sample scatterplot displaying the fourth component in the metabolomics and transcriptomics data set (upper diagonal plot) and Pearson correlation between the variates of the two omics blocks for component 4 (lower diagonal plot).

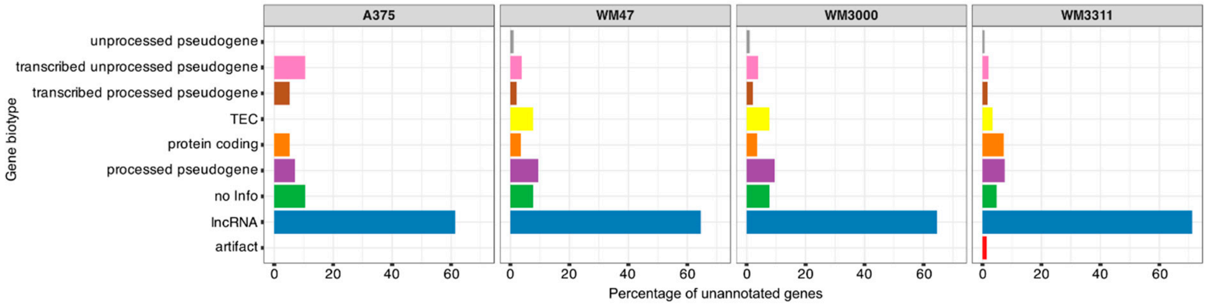

Supplementary Figure S2. Gene biotype composition of unannotated transcriptomic features. Since many detected transcripts lacked formal functional annotation, biotype metadata was assigned via Ensembl BioMart. Plots represent the proportional composition of these biotypes within the unannotated gene set for each of the four melanoma xenograft models (A375, WM47, WM3000, and WM3311). lncRNA: long non-coding RNA; TEC: To be experimentally confirmed.

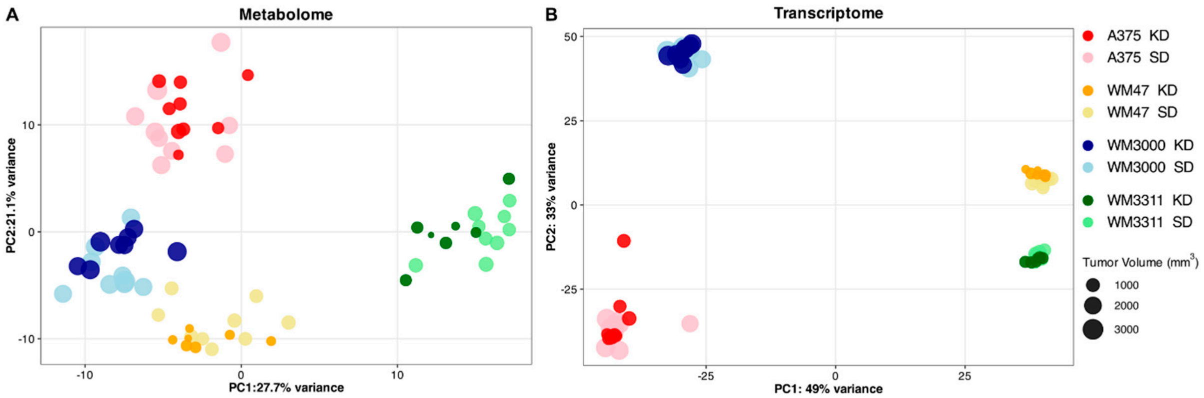

Supplementary Figure S3. Melanoma xenografts differ distinctly in their metabolome and transcriptome. (A, B) Principal component analysis (PCA) of the tumor (**A**) metabolome and (**B**) transcriptome derived from A375, WM47, WM3000, and WM3311 melanoma xenograft tumors treated with KD or SD. n = 7-9 per group. Dot size represents the tumor volume at the time of harvest.

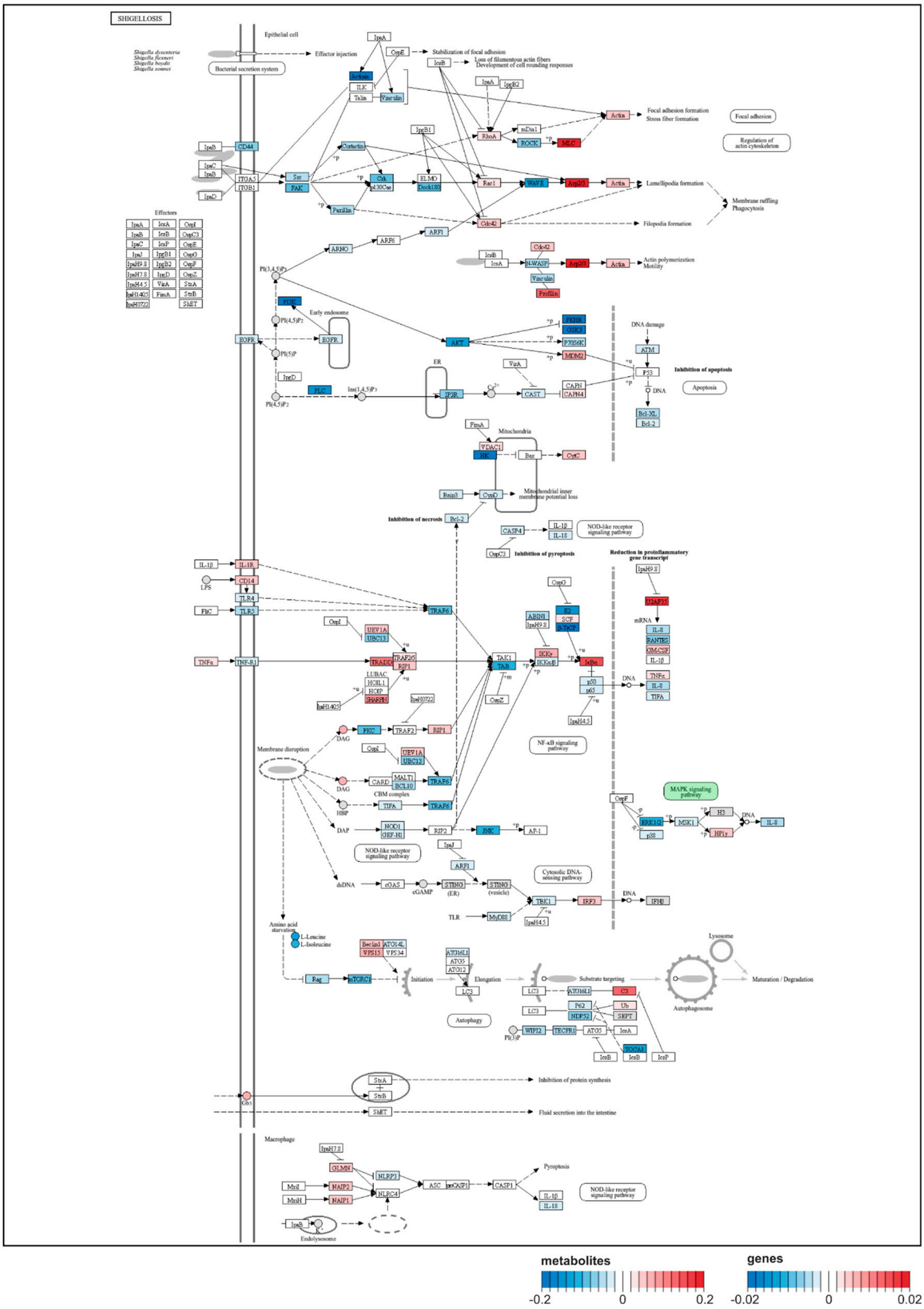

Supplementary Figure S4. Integration of metabolomic and transcriptomic DIABLO loadings within the human Shigellosis pathway. Pathview-generated KEGG map (hsa05131, retrieved from www.kegg.jp) for the human Shigellosis pathway, illustrating metabolite and gene loadings from the DIABLO analysis. The color of each node corresponds to its loading value, as indicated by the accompanying color legend (blue for negative loadings indicating KD-induced downregulation; red for positive loadings indicating KD-induced upregulation). For KEGG nodes representing multiple genes, the color reflects the sum of their individual loading values. For metabolite classes with multiple features, the color represents the maximum absolute loading value among its features.

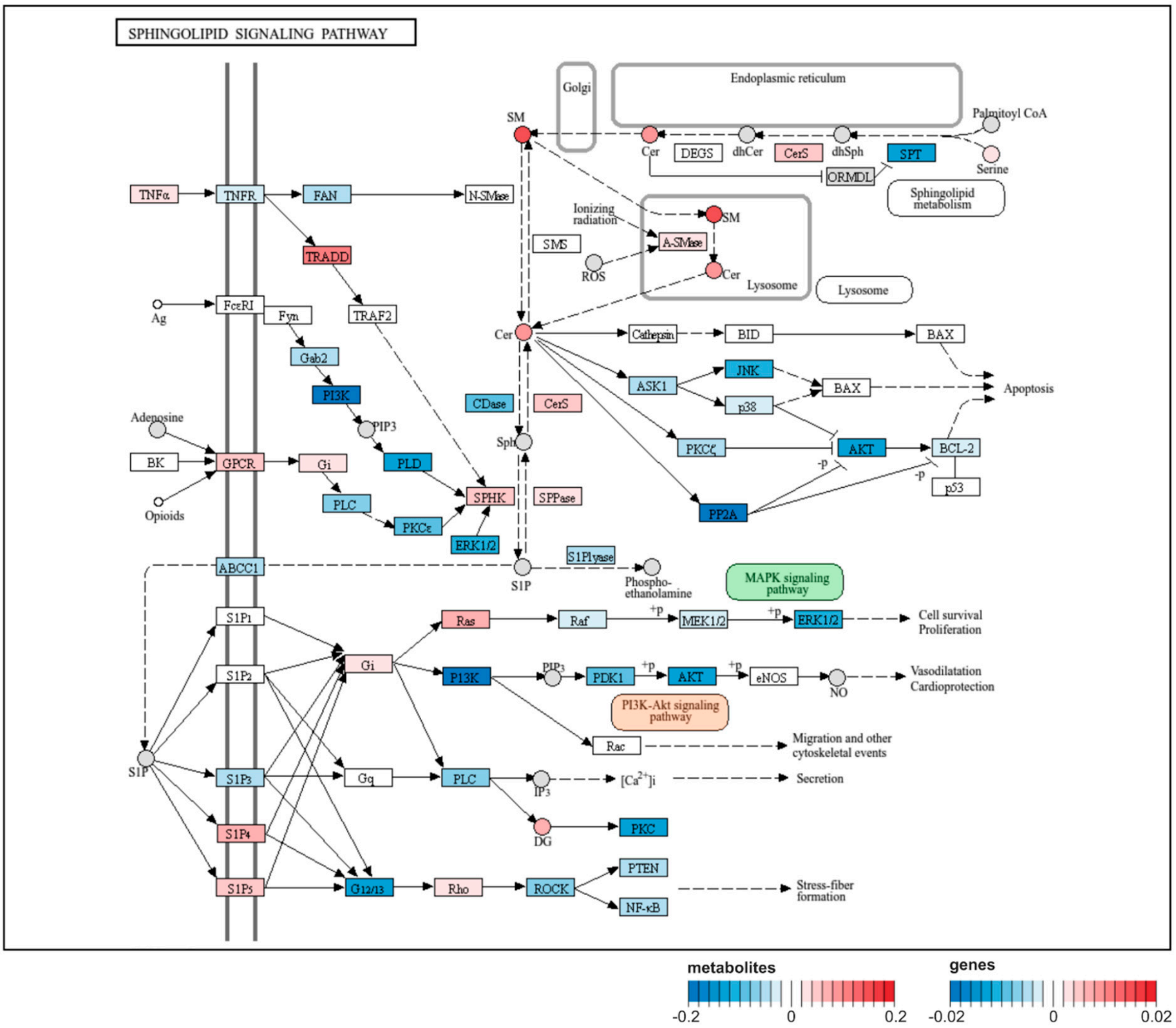

Supplementary Figure S5. Integration of metabolomic and transcriptomic DIABLO loadings within the human Sphingolipid signaling pathway. Pathview-generated KEGG map (hsa04071, retrieved from www.kegg.jp) for the human sphingolipid signaling pathway, illustrating metabolite and gene loadings from the DIABLO analysis. The color of each node corresponds to its loading value, as indicated by the accompanying color legend (blue for negative loadings indicating KD-induced downregulation; red for positive loadings indicating KD-induced upregulation). For KEGG nodes representing multiple genes, the color reflects the sum of their individual loading values. For metabolite classes with multiple features, the color represents the maximum absolute loading value among its features.

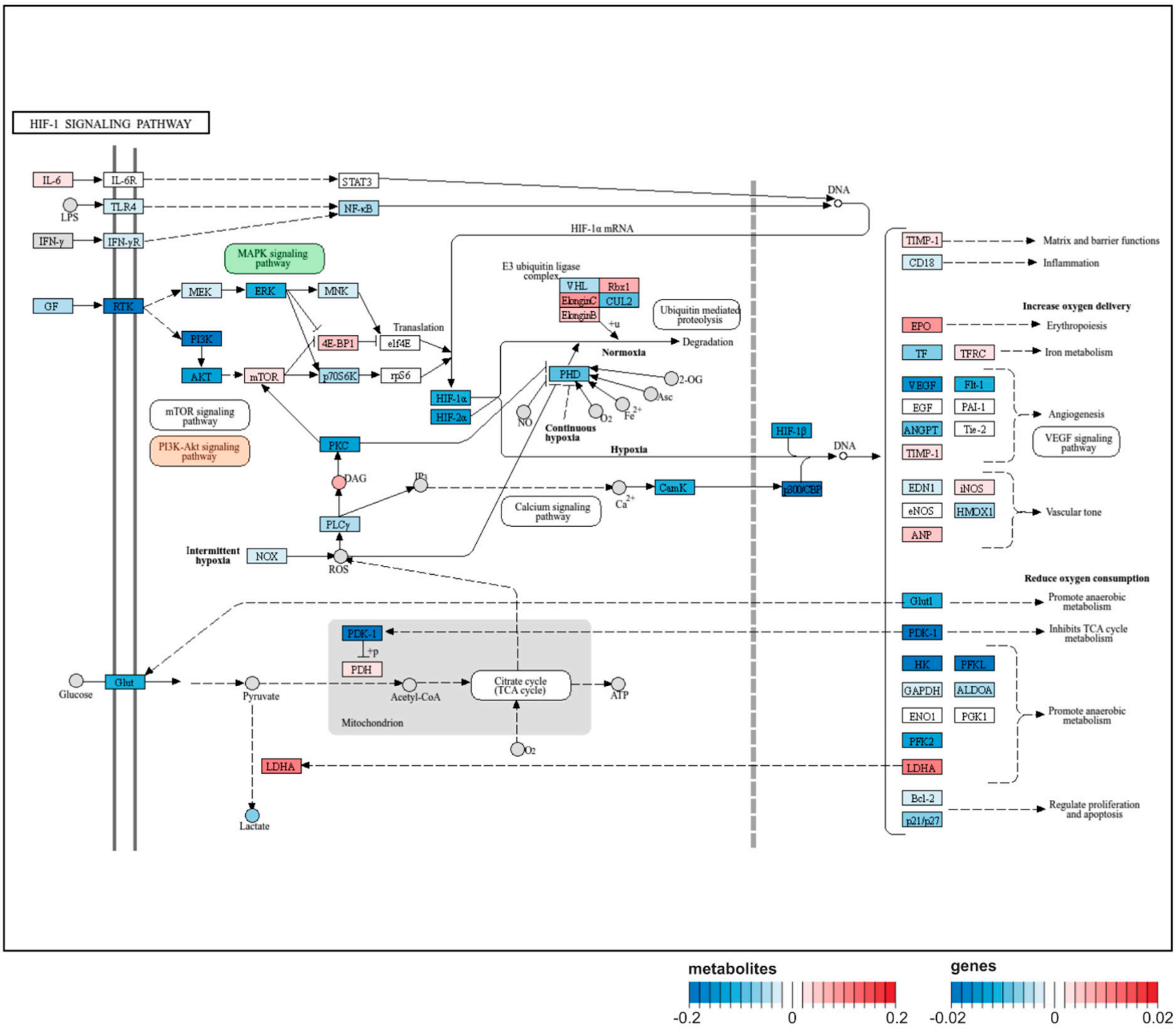

Supplementary Figure S6. Integration of metabolomic and transcriptomic DIABLO loadings within the human HIF-1 signaling pathway. Pathview-generated KEGG map (hsa04066, retrieved from www.kegg.jp) for the human HIF-1 signaling pathway, illustrating metabolite and gene loadings from the DIABLO analysis. The color of each node corresponds to its loading value, as indicated by the accompanying color legend (blue for negative loadings indicating KD-induced downregulation; red for positive loadings indicating KD-induced upregulation). For KEGG nodes representing multiple genes, the color reflects the sum of their individual loading values. For metabolite classes with multiple features, the color represents the maximum absolute loading value among its features.

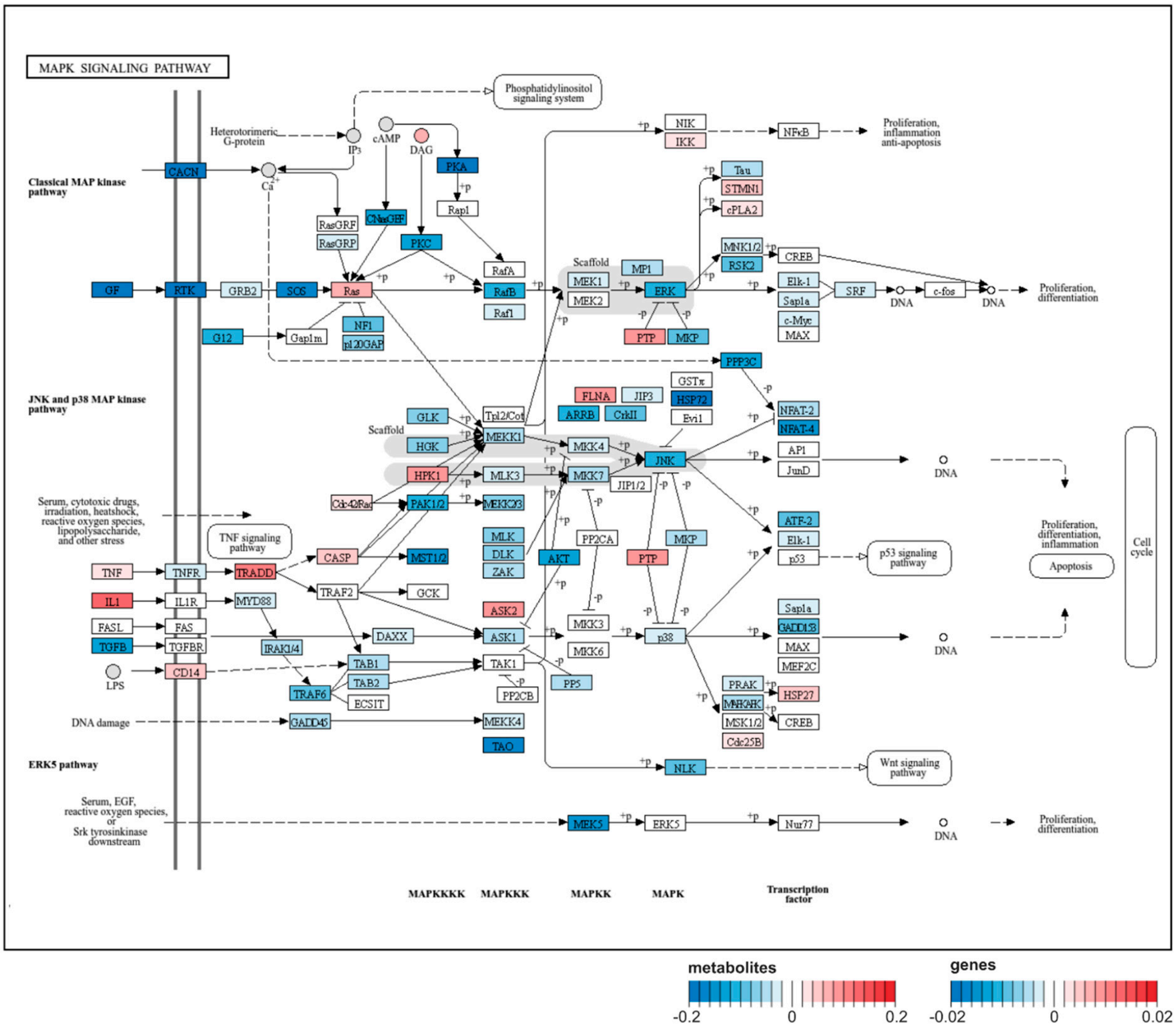

Supplementary Figure S7. Integration of metabolomic and transcriptomic DIABLO loadings within the human MAPK signaling pathway. Pathview-generated KEGG map (hsa04010, retrieved from www.kegg.jp) for the human MAPK signaling pathway, illustrating metabolite and gene loadings from the DIABLO analysis. The color of each node corresponds to its loading value, as indicated by the accompanying color legend (blue for negative loadings indicating KD-induced downregulation; red for positive loadings indicating KD-induced upregulation). For KEGG nodes representing multiple genes, the color reflects the sum of their individual loading values. For metabolite classes with multiple features, the color represents the maximum absolute loading value among its features.

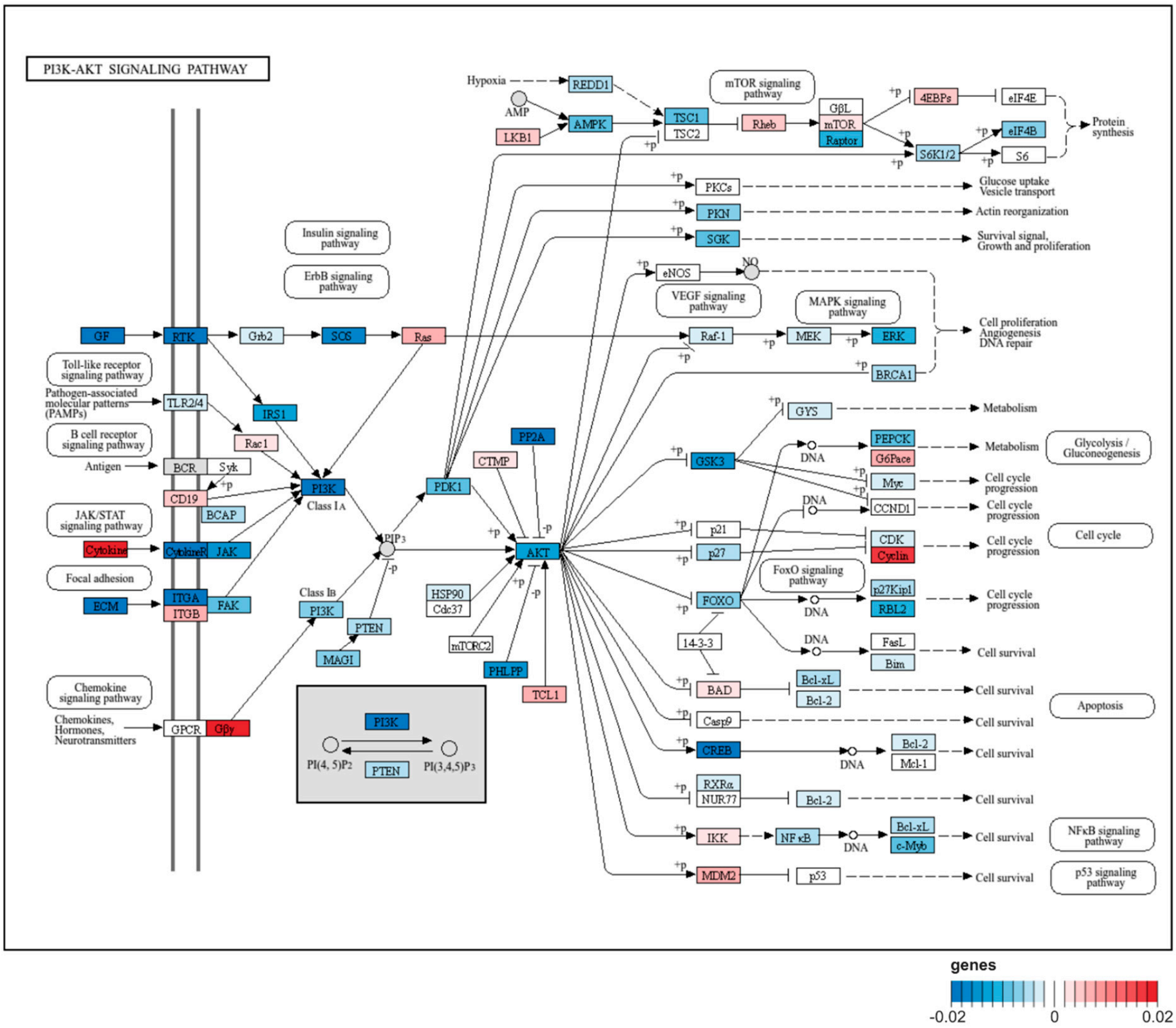

Supplementary Figure S8. Integration of metabolomic and transcriptomic DIABLO loadings within the human PI3K-Akt signaling pathway. Pathview-generated KEGG map (hsa04151, retrieved from www.kegg.jp) for the human PI3K-Akt signaling pathway, illustrating metabolite and gene loadings from the DIABLO analysis. The color of each node corresponds to its loading value, as indicated by the accompanying color legend (blue for negative loadings indicating KD-induced downregulation; red for positive loadings indicating KD-induced upregulation). For KEGG nodes representing multiple genes, the color reflects the sum of their individual loading values.
